# Endoplasmic reticulum stress stimulates the release of extracellular vesicles carrying danger-associated molecular pattern (DAMP) molecules

**DOI:** 10.18632/oncotarget.24158

**Published:** 2018-01-11

**Authors:** Gavin P. Collett, Christopher W. Redman, Ian L. Sargent, Manu Vatish

**Affiliations:** ^1^ Nuffield Department of Women's & Reproductive Health, University of Oxford, Women's Centre Level 3, John Radcliffe Hospital, Oxford OX3 9DU, UK

**Keywords:** endoplasmic reticulum stress, extracellular vesicles, ER stress, pathology

## Abstract

Disturbances in endoplasmic reticulum (ER) function lead to ER stress which, when severe or prolonged, may result in apoptosis. Severe ER stress has been implicated in several pathological conditions including pre-eclampsia, a multisystem disorder of pregnancy associated with the release of pro-inflammatory factors from the placenta into the maternal circulation. Here, we show that severe ER stress induced by two distinct mechanisms in BeWo choriocarcinoma cells leads to the release of extracellular vesicles (EVs) carrying pro-inflammatory damage-associated molecular pattern (DAMP) molecules. Co-treatment with the antioxidant pyrrolidine dithiocarbamate results in a reduction in ER stress-induced EV-associated DAMP release. We further demonstrate that severe ER stress is associated with changes in the expression of several stress-related proteins, notably Cited-2 and phosphorylated JNK. Together, these data indicate that severe ER stress-mediated release of EV-associated DAMPs may contribute to the heightened systemic maternal inflammatory response characteristic of pre-eclampsia and may also be relevant to other chronic inflammatory diseases which display elevated ER stress.

## INTRODUCTION

Endoplasmic reticulum stress, resulting from disturbances in ER function and the accumulation of unfolded or misfolded proteins in the ER lumen, leads to the unfolded protein response (UPR), a cytoprotective response comprising multiple intracellular signalling pathways [1]. Under mild ER stress conditions, activation of these pathways is sufficient to restore normal ER function and cellular homeostasis. However, if ER stress is severe or prolonged, or if UPR signalling pathways are compromised, then apoptotic cell death takes place. Chronic ER stress, with ensuing cell injury or death, has been implicated in a number of pathological conditions including diabetes [2], atherosclerosis [3] and neurodegenerative diseases [4]. In pregnancy, it has been shown that placentae from cases of intrauterine growth restriction (IUGR) display activation of ER stress pathways and that these are further elevated in IUGR pregnancies complicated with pre-eclampsia (PE), a multisystem disorder of pregnancy characterised by an exacerbated maternal systemic inflammatory response [5]. More recently, it was revealed that activation of placental ER stress response pathways is greater in severe early-onset PE (<34 weeks) than in late-onset disease (≥34 weeks) [6].

Pre-eclampsia is associated with the release of multiple pro-inflammatory factors from the placental syncytiotrophoblast into the maternal circulation. These factors include extracellular vesicles (EVs) and damage associated molecular pattern molecules (DAMPs). EVs comprise exosomes (~50-200 nm, released by exocytosis of endosome-derived multivesicular bodies), microvesicles (~100 nm-1μm, released by direct budding from the plasma membrane) and apoptotic bodies (~1-5μm, released by cells undergoing programmed cell death) [7], and have been implicated in both the maintenance of normal pregnancy and the pathology of disorders of pregnancy, particularly pre-eclampsia [8]. DAMPs, also known as alarmins, are highly pro-inflammatory molecules released by stressed or damaged cells which act as endogenous danger signals [9]. They can bind to and activate cells of the innate immune system leading to the initiation or exacerbation of a pro-inflammatory response. Examples of DAMPs include high mobility group box-1 (HMGB1), heat shock proteins, S100 calcium-binding proteins, histones and extracellular ATP. Increased serum levels of DAMPs have been associated with inflammatory diseases such as systemic lupus erythematosus [10], atherosclerosis [11] and acute pancreatitis [12]. A role for DAMPS in the pathophysiology of pre-eclampsia is supported by the observation that women with pre-eclampsia have increased serum levels of several DAMPs including HMGB1 [13], HSP70 [14] and S100B [15]. Furthermore, both HMGB1 [16] and S100B [17] have been shown to be released from trophoblast cells in response to oxidative stress.

Although DAMPs are primarily considered to be soluble molecules, there is growing interest in the notion that DAMP-carrying EVs released from stressed or injured tissues may play a role in the induction or persistence of inflammation [18]. HMGB1 [19], heat shock proteins [20] and ATP [21] have all been shown to be carried in EVs. However, the biological role of EV-associated DAMPs is yet to be ascertained. Whilst it has been demonstrated that palmitate-induced ER stress in hepatocytes stimulates the release of EVs [22], it is unclear whether ER stress can lead to the release of EVs carrying DAMPs. In this study we show that severe ER stress generated by two distinct pathways in BeWo choriocarcinoma cells (a cell line used to model trophoblast) results in the release of EV-associated DAMPs. We propose that this is a mechanism which may contribute to the clinical symptoms of pre-eclampsia and other chronic inflammatory diseases.

## RESULTS

### Induction of mild and severe ER stress by tunicamycin in BeWo cells

**Figure 1:**
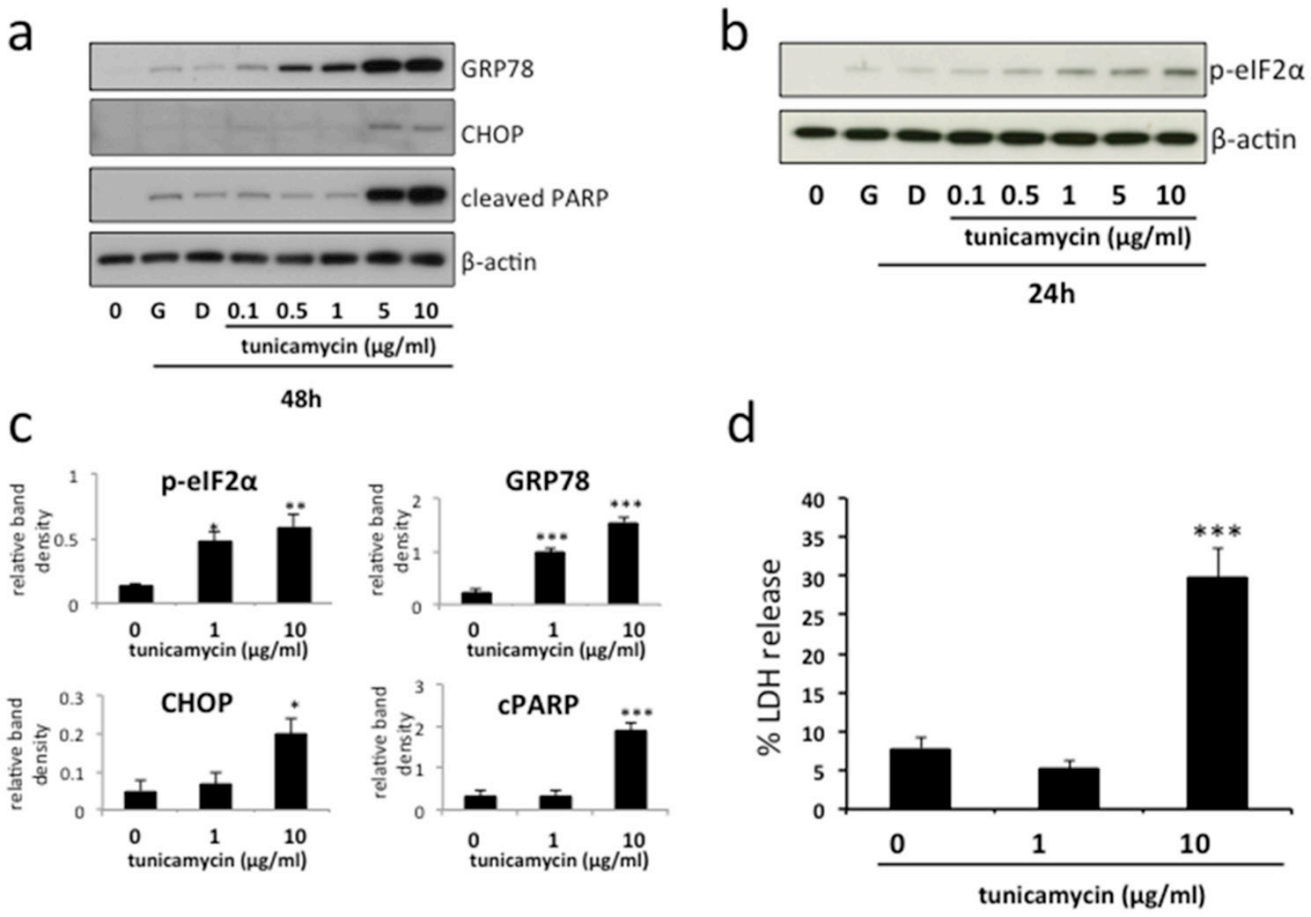
Induction of mild and severe ER stress by tunicamycin in BeWo cells BeWo cells were treated with growth medium alone (G), vehicle (DMSO) or the indicated concentrations of tunicamycin for 48h **(a)** or 24h **(b)**. Cell lysates were analysed for the expression of GRP78, phospho-eIF2α, CHOP, cleaved PARP and β-actin by immunoblotting. Representative blots from three separate experiments are shown. **(c)** Blots were analysed by densitometric image analysis (Image J), normalising to β-actin, for GRP78, phospho-eIF2α, CHOP & cleaved PARP. Results are represented as mean ± SEM for three separate experiments. ^*^p<0.05, ^**^p<0.01, ^***^p<0.001 compared with control (ANOVA). **(d)** Cells were treated with 1μg/ml or 10μg/ml tunicamycin for 48h, representing mild and severe ER stress respectively. Cell death was analysed by release of lactate dehydrogenase (LDH) into the culture medium. Results are represented as mean ± SEM for four separate experiments. ^***^p<0.001 compared with control (ANOVA).

**Figure 2:**
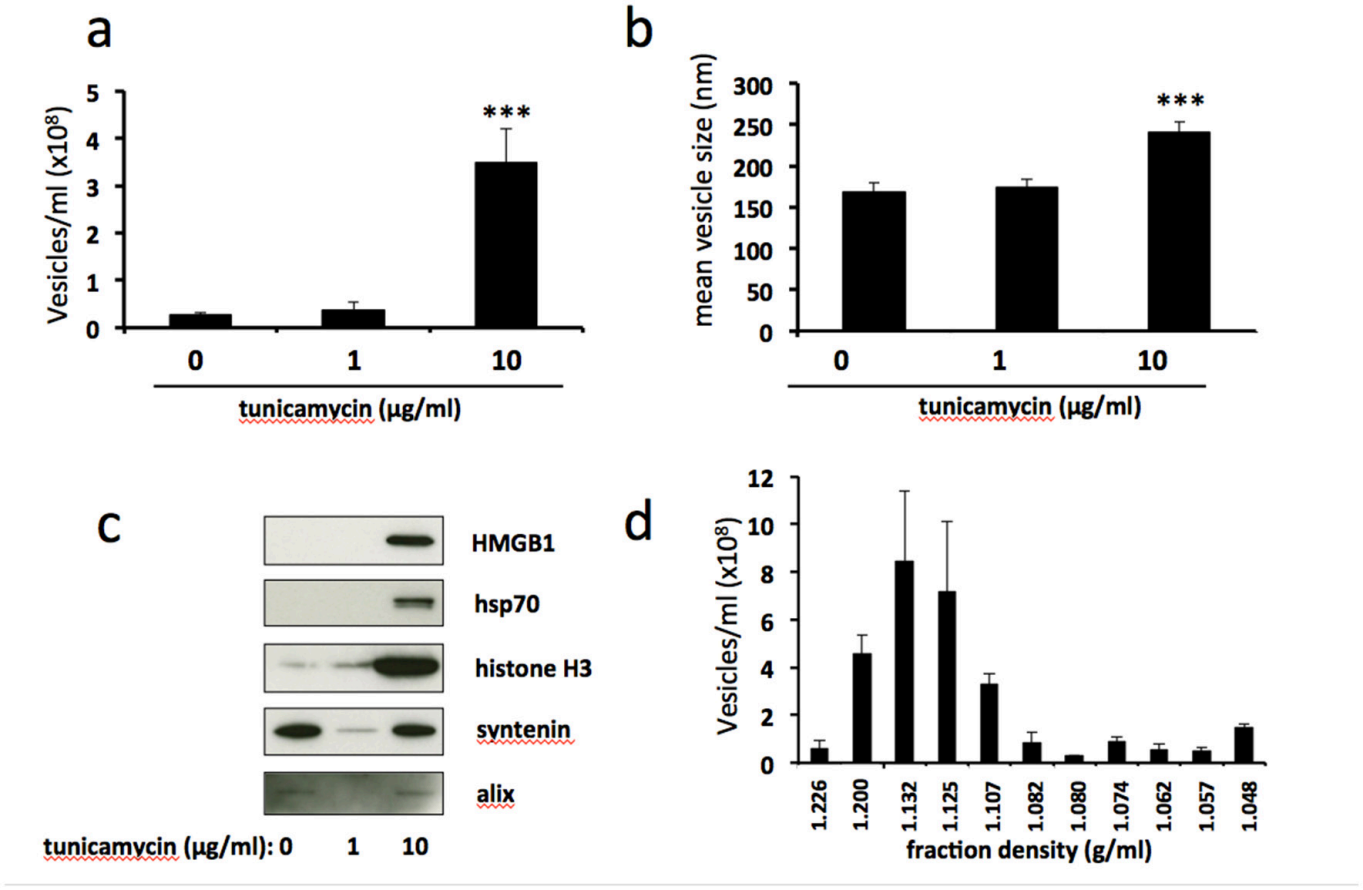
Severe ER stress stimulates the release of extracellular vesicles carrying danger-associated molecular pattern (DAMP) molecules BeWo cells were treated with 1μg/ml or 10μg/ml tunicamycin, or DMSO control, for 48h. Extracellular vesicles were isolated from the culture medium by ultracentrifugation and analysed by NTA for quantity **(a)** and size **(b)**. Equal volumes of EV pellet suspensions were analysed for expression of DAMPs HMGB1, hsp70 and histone H3, and exosome markers syntenin and alix by immunoblotting **(c)**. **(d)** EV pellets from severely ER-stressed cells were resuspended in PBS (500μl) and overlaid onto 5-40% discontinuous iodixanol gradients. Ultracentrifugation was carried out at 150,000x*g* (maximum) for 18 h at 4°C. Fractions (12 × 1ml) were collected manually and EV concentrations were analysed by NTA. Results are represented as mean ± SEM for six (a-c) or three (d) separate experiments. ^***^p<0.001 compared with control (ANOVA).

Mild ER stress activates the unfolded protein response. However, if it is severe or prolonged ER stress results in cell death. To mimic these effects *in vitro*, we treated BeWo cells with increasing doses of tunicamycin, a compound routinely used to induce ER stress (Figure 1a). Immunoblotting revealed a dose-dependent upregulation of the chaperone protein GRP78 and phosphorylated eIF2α, signifying induction of an ER stress response, but only higher doses (5-10μg/ml) resulted in increased CHOP expression and PARP cleavage, signifying induction of more severe ER stress resulting in cell death (Figures 1a & 2a). Accordingly, for further experiments, tunicamycin concentrations of 1 and 10μg/ml were therefore selected to represent conditions of mild (significant upregulation of GRP78 & phosphorylated eIF2α but not CHOP & cleaved PARP, Figure 1c) and severe (significant upregulation of GRP78, phosphorylated eIF2α, CHOP & cleaved PARP, Figure 1c) ER stress respectively. Using these conditions we also observed significant increase of LDH release into the culture medium following severe ER stress induction, confirming the induction of cell death in response to severe, but not mild, ER stress (Figure 1d).

### Severe ER stress stimulates the release of extracellular vesicles carrying danger-associated molecular pattern (DAMP) molecules

To determine the effect of ER stress on EV release we subjected BeWo cells to mild or severe ER stress and assessed EV number and size by nanoparticle tracking analysis. This revealed a significant increase in the number of EVs released in response to severe, but not mild, ER stress (Figure 2a). Furthermore, the mean size of vesicles released from severely ER-stressed cells was significantly greater than those shed from unstressed or mildly ER-stressed cells (Figure 2b). Assessment of isolated EVs for expression of damage-associated molecular patterns (DAMPs) by immunoblotting revealed high levels of EV-associated high mobility group protein B1 (HMGB1), heat shock protein 70 (hsp70) and histone H3 released from severely ER-stressed cells whereas these were low or undetectable from unstressed or mildly ER-stressed cells (Figure 2c). In contrast, expression of exosome markers syntenin and alix was detectable in EVs released from unstressed cells but was not increased in response to severe ER stress. It should be noted that these immunoblots were designed to detect the relative amounts of EV-associated DAMPs released into the culture medium and therefore equal volumes of the resuspended EV pellets were loaded. Since these would be expected to contain different amounts of protein (due to differing numbers of EV present) a loading control was not used. Further characterisation of EVs released from severely ER-stressed cells was carried out using iodixanol density gradient centrifugation followed by NTA. In iodixanol gradients exosomes localise to fractions with a buoyant density of 1.09-1.11g/ml [23, 24]. We found only a small proportion (~11%) of EVs in the fraction corresponding to this density (Figure 2d). The majority of EVs localised to fractions with higher densities (1.13-1.2), indicating a predominance of microvesicles and/or apoptotic bodies.

### Antioxidant treatment attenuates ER stress-induced release of extracellular vesicles

**Figure 3:**
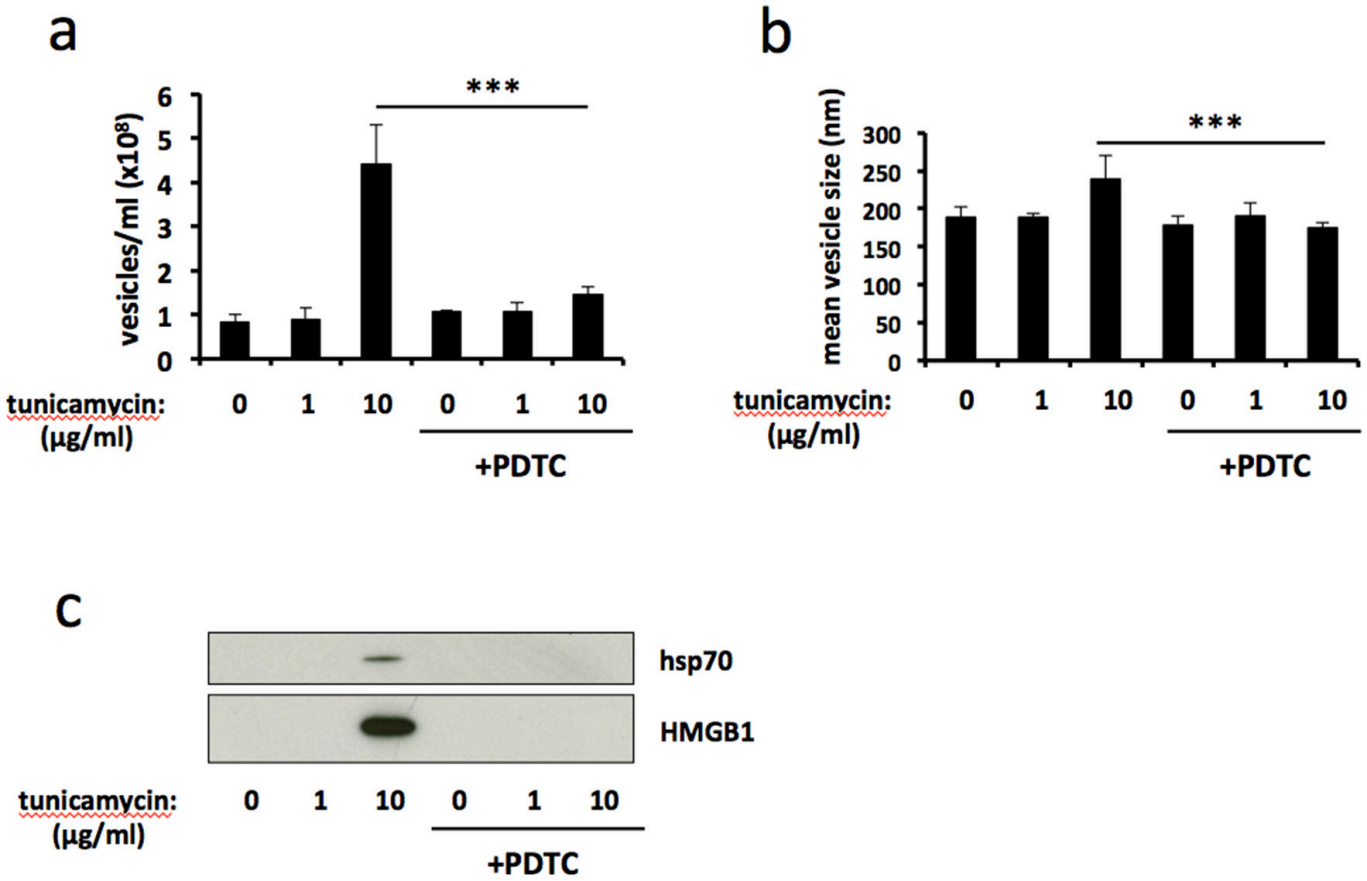
Antioxidant treatment attenuates ER stress-induced release of extracellular vesicles BeWo cells were treated with 1μg/ml or 10μg/ml tunicamycin, or DMSO control, for 48h in the presence or absence of the ROS scavenging antioxidant pyrrolidine dithiocarbamate (PDTC). Extracellular vesicles were isolated from the culture medium by ultracentrifugation and analysed by NTA for quantity **(a)** and size **(b)**. Equal volumes of EV pellet suspensions were analysed for expression of HMGB1 and hsp70 by immunoblotting **(c)**. Results are represented as mean ± SEM for three separate experiments. ^***^p<0.001 compared with control (ANOVA).

ER stress is associated with increased production of reactive oxygen species (ROS) and the development of oxidative stress [25]. Therefore we used the ROS-scavenging antioxidant pyrrolidine dithiocarbamate (PDTC) to examine the role of oxidative stress on ER stress-induced release of EVs. Treatment with PDTC significantly reduced the number of EVs released in response to severe ER stress (Figure 3a), and reversed the ER-stress induced increase in mean vesicle size (Figure 3b). Furthermore, release of vesicle-associated HMGB1 and hsp70 was completely abolished by PDTC treatment (Figure 3c). Taken together, these results show that antioxidant treatment attenuates ER stress-induced release of EVs.

### Thapsigargin-induced severe ER stress stimulates release of EV-associated DAMPs

**Figure 4:**
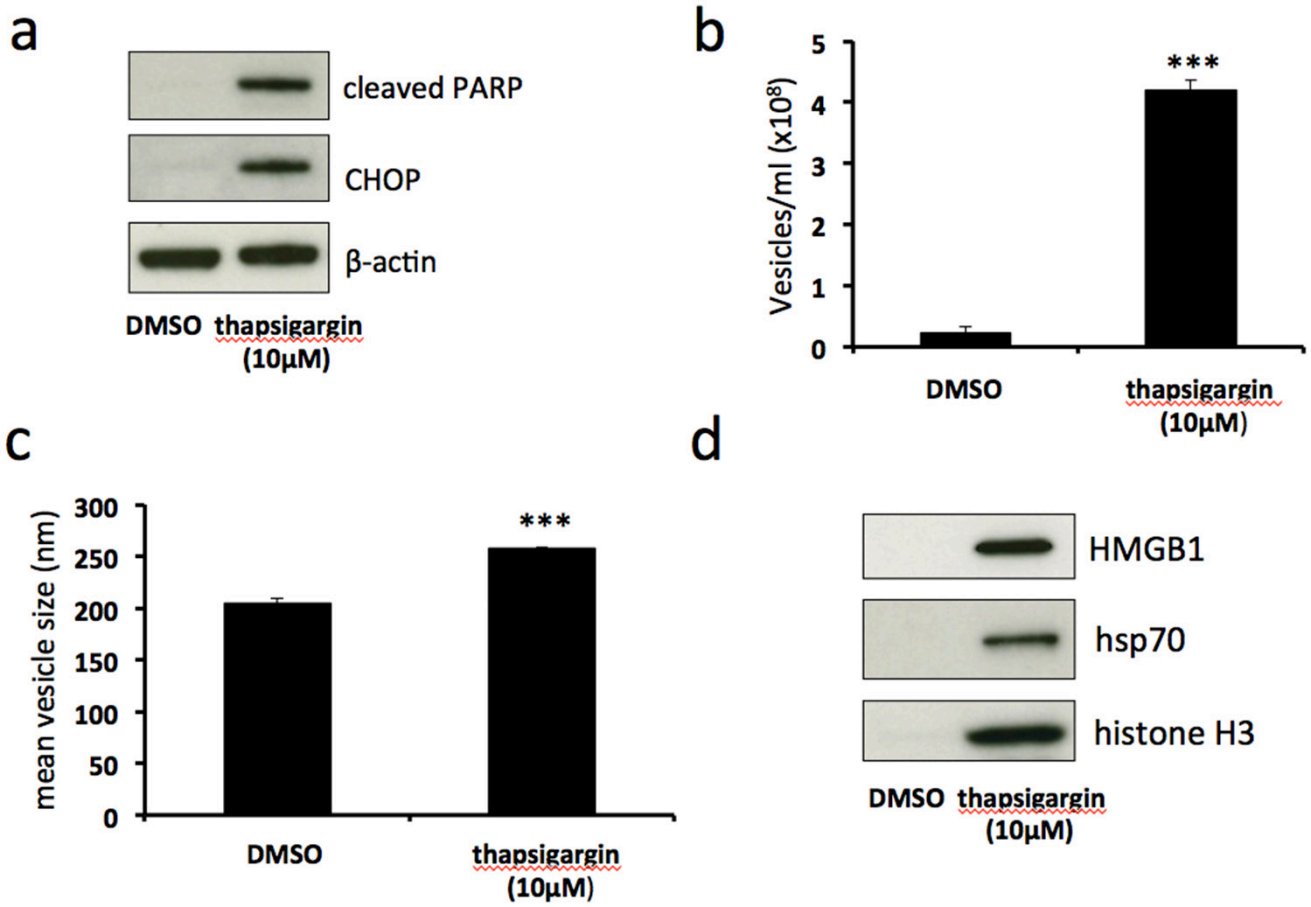
Thapsigargin-induced severe ER stress stimulates release of EV-associated DAMPs BeWo cells were treated with 10μM thapsigargin or DMSO control for 48h. Cell lysates were analysed for the expression of CHOP, cleaved PARP and β-actin by immunoblotting **(a)**. Extracellular vesicles were isolated from the culture medium by ultracentrifugation and analysed by NTA for quantity **(b)** and size **(c)**. Equal volumes of EV pellet suspensions were analysed for expression of HMGB1, hsp70 and histone H3 by immunoblotting **(d)**. Results are represented as mean ± SEM for three separate experiments. ^***^p<0.001 compared with control (Students t test).

Tunicamycin induces ER stress by inhibiting N-linked glycosylation, leading to an accumulation of unfolded or misfolded proteins in the ER lumen. In order to assess whether ER stress induced by a different mechanism results in similar release of EV-associated DAMPs, we used thapsigargin, a compound which induces ER stress by disruption of calcium homeostasis. Treatment of BeWo cells with 10μM thapsigargin resulted in upregulation of CHOP expression and the induction of PARP cleavage (Figure 4a). This was accompanied by a significant increase in both number (Figure 4b) and size (Figure 4c) of shed EVs. Immunoblotting revealed that these EVs carried DAMPs HMGB1, hsp70 and histone H3 (Figure 4d). These results show that, as does tunicamycin treatment, severe ER stress induced by thapsigargin stimulates the release of EV-associated DAMPs.

### Regulation of stress-related proteins during ER stress

**Figure 5:**
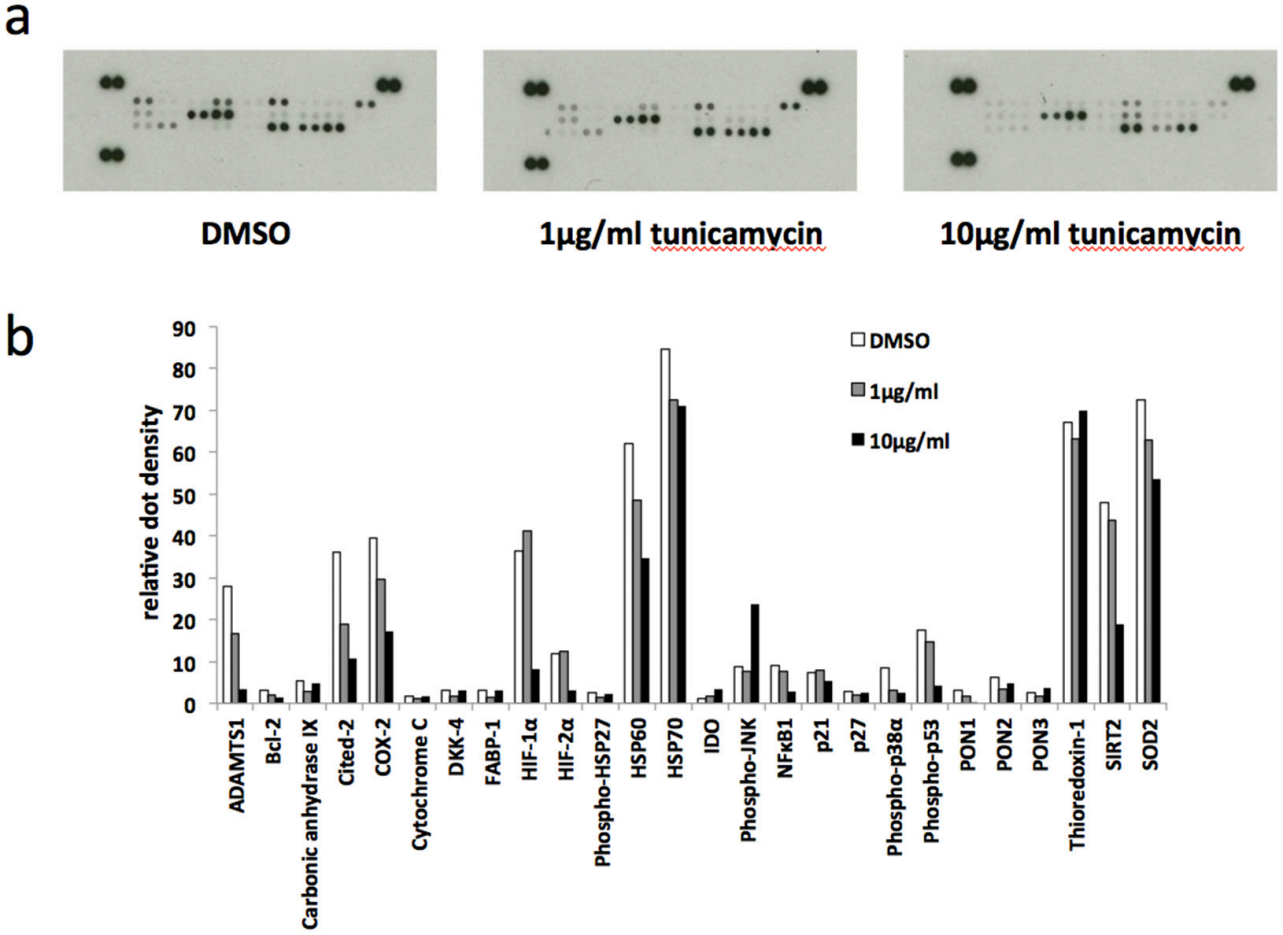
Human cell stress arrays BeWo cells were treated with 1μg/ml or 10μg/ml tunicamycin, or DMSO control, for 48h. **(a)** Cell lysate samples containing equal amounts of protein were analysed on human cell stress arrays. **(b)** The blots shown in (A) were analysed by densitometric image analysis (Image J). The graph displays the proteins in the order on the dot blots, from top left to bottom right excluding the three positive control pairs of dots on the top left, top right and bottom left.

**Figure 6:**
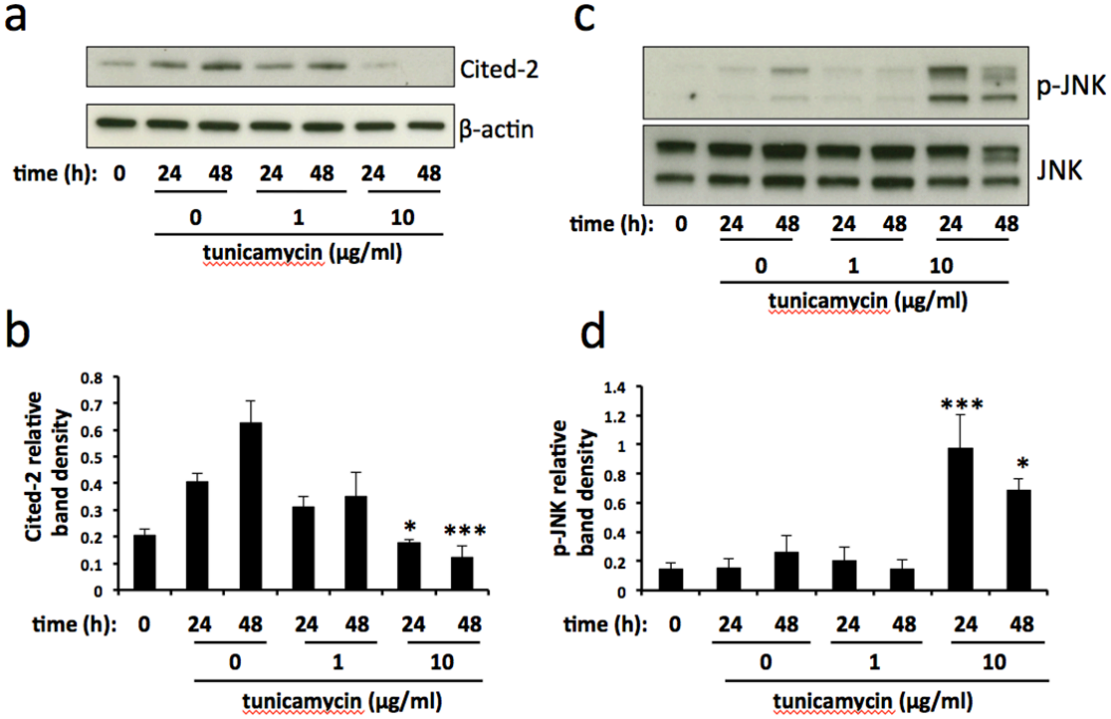
Cited-2 and phospho-JNK expression is altered in severe ER stress BeWo cells were treated with 1μg/ml or 10μg/ml tunicamycin, or DMSO control, for 24h and 48h. At each time point cell lysates were made and analysed for the expression of Cited-2 **(a)** and phospho-JNK **(c)**. Blots were analysed by densitometric image analysis (Image J), normalising to β-actin for Cited-2 **(b)** and total JNK for phospho-JNK **(d)**. Results are represented as mean ± SEM for three separate experiments. ^*^p<0.05, ^***^p<0.001 compared with control (ANOVA).

In order to gain an insight into the pathways involved in ER stress-stimulated EV release in BeWo cells we used a human cell stress array. The expression of 26 cell stress-related proteins was analysed in lysates prepared from BeWo cells subjected to tunicamycin-induced mild or severe ER stress, or DMSO control, for 48h (Figure 5a). Although not quantitative, densitometric analysis of the developed arrays indicates trends of protein expression in response to ER stress (Figure 5b). Several proteins were downregulated in response to severe ER stress, most notably ADAMTS1, Cox-2, Cited-2, HIF-1α and SIRT2. One protein, phospho-JNK, was upregulated by severe ER stress. We then used immunoblotting to validate the changes in expression of two of these proteins, Cited-2 and phospho-JNK. In these experiments lysates were made at 24h as well as 48h. Cited-2 was significantly downregulated by severe ER stress at both 24h and 48h compared with vehicle control (Figures 6a & 6b). Mild ER stress had no significant effect on Cited-2 expression. Phospho-JNK levels were significantly increased by severe ER stress at both 24h and 48h but unchanged by mild ER stress (Figures 6c & 6d). These results suggest that downregulation of Cited-2 expression and increased JNK phosphorylation may be involved in the mechanism of ER stress-induced release of EV-associated DAMPs.

## DISCUSSION

**Figure 7:**
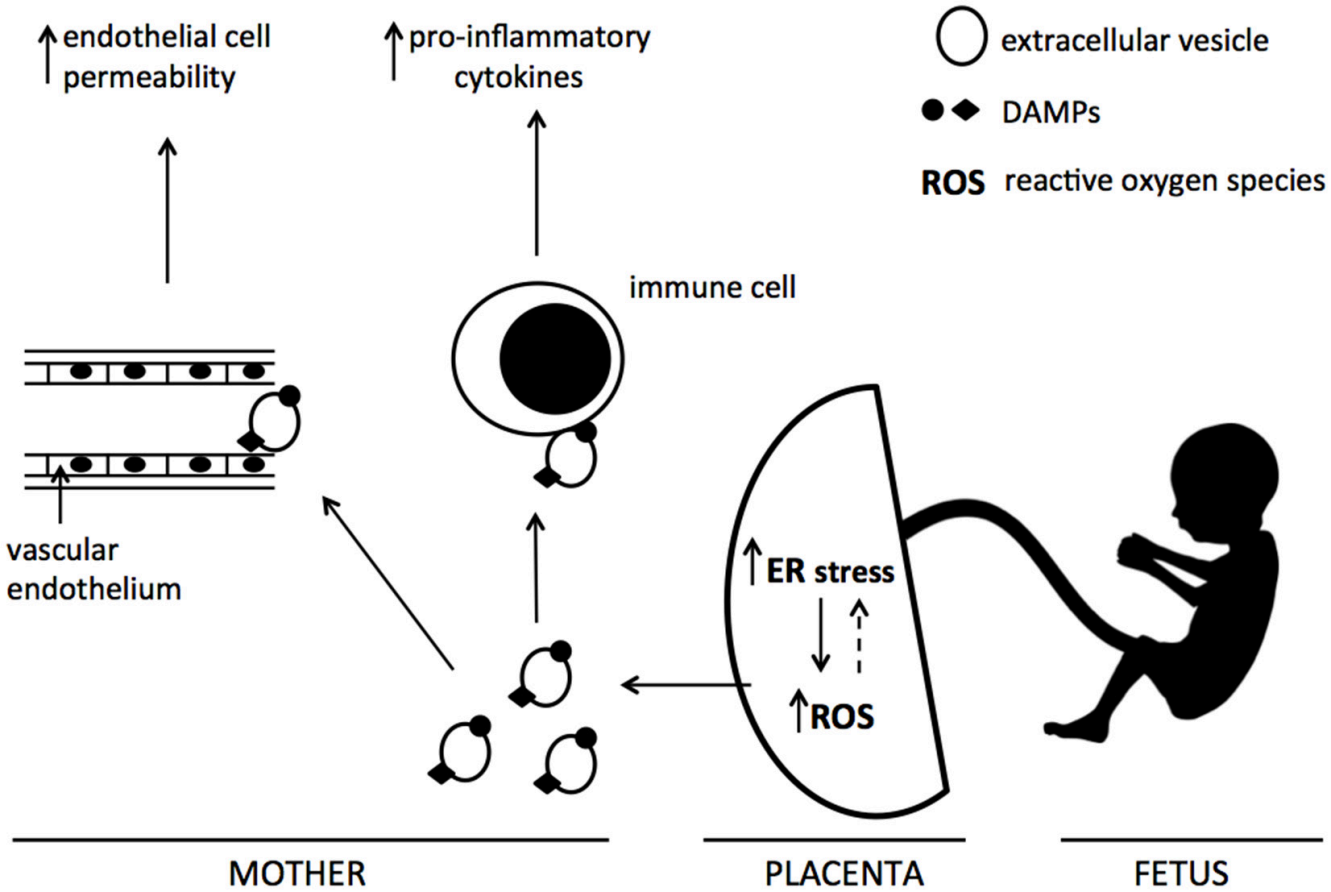
Proposed mechanism of action of ER stress-induced EV-associated DAMPs Increased placental ER stress, via the production of reactive oxygen species, leads to the release of extracellular vesicles carrying DAMPs into the maternal circulation. These EV-associated DAMPs may interact with maternal endothelial and immune cells, resulting in exacerbated endothelial cell permeability and pro-inflammatory cytokine production.

In this study we demonstrate for the first time that severe ER stress leads to the release of EV-associated DAMPs from BeWo cells. We propose that their release from the placenta through this mechanism may contribute to the exacerbated maternal systemic inflammatory response characteristic of pre-eclampsia (Figure 7).

We have used BeWo choriocarcinoma cells as a model of human trophoblast in this study. Although trophoblast cell lines have some limitations, BeWo cells have been used in previous studies to investigate the release of trophoblast-derived EVs [26, 27]. Furthermore, isolation of primary cytotrophoblasts from placental tissue is associated with the activation of ER stress pathways (G Collett, unpublished observations) which may render these cells inappropriate for the investigations described here. Similarly, we chose to use non-syncytialised BeWo cells for this study since syncytialisation of these cells in culture is associated with decreased cell viability and apoptosis [28] and the activation of ER stress pathways (G Collett, unpublished observations).

Pre-eclampsia is associated with increased placental stress, including activation of ER stress pathways. We have demonstrated that severe ER stress, induced by two different mechanisms and resulting in CHOP upregulation and cell death, results in the production of EVs carrying pro-inflammatory DAMPs, whereas mild ER stress does not. These findings are consistent with the hypothesis that low levels of placental ER stress result in reduced cell proliferation and a growth restricted phenotype whereas higher levels of ER stress lead to activation of pro-inflammatory pathways characteristic of pre-eclampsia [29]. Increased shedding of EVs as a result of cellular stress has been reported previously. For example, oxidative stress increases exosome secretion from retinal pigment epithelial cells whilst adipocytes exposed to lipotoxic stress show enhanced release of extracellular vesicles [30]. Furthermore, ER stress stimulates the release of pro-inflammatory EVs from hepatocytes [22]. Our results show that, in common with most cell types, unstressed BeWo cells constitutively release exosomes that express exosome markers alix and syntenin and carry low or undetectable levels of DAMPs. However, following severe ER stress the observed increase in released EVs is not accompanied by a concomitant increase in EV-associated alix and syntenin expression. Thus it is likely that the EVs shed in response to severe ER stress are predominantly microvesicles and/or apoptotic bodies. This is supported by two further findings: only a small minority of EVs from severely ER-stressed cells localise during density gradient centrifugation to fractions with a buoyant density characteristic of exosomes, and the mean size of EVs released by severely ER-stressed cells is significantly larger than those released by unstressed cells. In addition these vesicles carry high levels of DAMPs HMGB1, hsp70 and histone H3. Increased release of DAMPs has been implicated in the pathogenesis of pre-eclampsia. Circulating levels of HMGB1 [13, 31], hsp70 [32], extracellular ATP [33], S100B [15, 34] and mitochondrial DNA [35] are significantly elevated in women with pre-eclampsia whilst HMGB1 mRNA and protein expression are increased in severe pre-eclamptic placentas [33]. In addition, HMGB1 released from cultured trophoblast cells following hypoxic stress increases endothelial cell permeability, a feature of pre-eclampsia [16]. Extensive research has highlighted the role of DAMPs in sterile inflammation but it is becoming clear that these molecules can be released from cells in or on EVs as well as in soluble form. For example, HMGB1 is highly concentrated within apoptotic vesicles [19] whilst hsp70 has been shown to be released in a vesicular form which can activate macrophages [36]. Whether EV-associated DAMPs have different functions to those released in soluble form remains to be determined, although vesicular hsp70 is at least 260-fold more effective in inducing TNF-alpha production from macrophages than free recombinant hsp70 [36]. Interestingly, release of DAMPs, including histones and hsp family members, from apoptotic bodies through limited membrane permeabilisation prior to secondary necrosis has been observed, raising the possibility of localised release of pro-inflammatory factors by this EV-mediated mechanism during the early phase of apoptosis [37].

The molecular mechanism by which ER stress leads to the release of EV-associated DAMPs is yet to be elucidated. The endoplasmic reticulum maintains an oxidising environment which facilitates disulphide bond formation and normal protein folding [38]. However, ER stress is associated with the excessive production of reactive oxygen species (ROS) within the ER lumen, leading to oxidative stress and subsequent cell injury and/or death [39]. In accordance with this, our results using a ROS scavenging antioxidant, PDTC, showed complete abrogation of severe ER stress-induced EV release, suggesting that these effects of ER stress are mediated through the generation of ROS and oxidative stress. However, these results may not necessarily reflect a simple, linear upregulation of ROS following ER stress since the relationship between ER and oxidative stress is complex, with ROS playing a role both upstream and downstream of UPR signalling [27], potentially through the establishment of a positive feedback loop [40]. For further mechanistic investigation, we used a human cell stress array to identify proteins showing expression changes in cells undergoing severe ER stress. Cited-2 (CBP/p300-interacting transactivator with Glu/Asp-rich carboxy-terminal domain 2), a multifunctional transcriptional co-regulator with antiapoptotic properties [41], was significantly downregulated in severely ER stressed cells. A crucial role for Cited-2 in placental function is demonstrated by work showing that its loss in mouse syncytiotrophoblasts results in placental malformation and significantly reduced embryonic growth [42], although whether induction of ER stress plays a role in this is not known. Repression of Cited-2 expression has been shown to induce ER stress in neural stem cells [43] via a mechanism involving upregulation of miR-200b. Therefore, it is tempting to speculate that downregulation of this protein may be a key player in the induction of ER stress and subsequent release of EVs from trophoblast cells. However, it should be noted that Cited-2 expression is increased in culture under control (vehicle only) conditions. This may be due to increasing cell density, which has been shown to alter the expression of other proteins in culture [44, 45]. Thus it is possible that the growth inhibitory effect of ER stress may be responsible for the relative downregulation of Cited-2 expression compared with control; therefore we cannot rule out the possibility that Cited-2 may itself be regulated by ER stress rather than vice versa. These themes will be explored in future work.

Whilst most proteins showing a change in expression in ER stressed cells were downregulated, we found a significant elevation of phosphorylated JNK levels. JNK, which is activated by phosphorylation, has been shown to regulate the transition from adaptive to CHOP-dependent apoptotic UPR [46]. This is in agreement with our finding that severe, but not mild, ER stress resulted in a significant increase in JNK phosphorylation. JNK has been implicated in the release of HMGB1 [47]. In particular, JNK mediates free cholesterol-induced apoptosis, necrosis and secretion of HMGB1 in hepatocytes [48]. Thus it is likely that activation of JNK plays a role in the severe ER stress-induced apoptosis and release of EV-associated HMGB1 and other DAMPS which we have observed in this study.

In conclusion, our results suggest that severe ER stress-mediated release of EV-associated DAMPs may possibly contribute to the heightened systemic maternal inflammatory response and endothelial cell permeability characteristic of pre-eclampsia. These results may also be pertinent to other chronic inflammatory diseases which show elevated ER stress. Multiple mechanisms have been demonstrated by which ER stress can promote inflammation in these conditions, including delivery of danger signals to antigen presenting cells following ER stress-induced apoptosis [49]. Therefore it is conceivable that these effects may, in part, be mediated through the release of EV-associated DAMPs.

## MATERIALS AND METHODS

### Cell culture

BeWo cells were obtained from the European Collection of Cell Cultures (Porton Down, UK) and cultured in full growth medium (Dulbecco's modified Eagle's medium/Ham's F12 supplemented with 2 mM l-glutamine, 100 IU/ml penicillin, 100 μg/ml streptomycin (Sigma) and 10% (v/v) fetal calf serum (Serum Laboratories International)). Cells were grown as a monolayer at a density of 10^7^ cells per 75 mm^2^ flask at 37 °C in 95% air and 5% CO_2_, with medium changed every 48 h. For passages, cells were detached with trypsin/EDTA (Life Technologies) at 37 °C, then washed in complete culture medium and replated.

### Induction of ER stress and isolation of extracellular vesicles

Confluent T75 flasks of BeWo cells were treated with either growth medium containing tunicamycin (Sigma) or thapsigargin (Sigma) at the concentrations shown. DMSO was used as vehicle-only control. All media were made with FBS which had been centrifuged at 150,000x*g* overnight for depletion of extracellular vesicles. After 48h the medium was collected from the cells and spun at 1500x*g* for 10 min at 4°C to remove large cellular debris. The supernatant was spun for 1 h at 150,000x*g* at 4°C. The resultant EV pellet was then resuspended in sterile PBS for further analysis.

### Nanoparticle tracking analysis

EV sizes and concentrations were measured using the NanoSight NS500 instrument equipped with a 405 nm laser (Malvern, UK), sCMOS camera and nanoparticle tracking analysis (NTA) software version 2.3, Build 0033 (Malvern, UK) as previously described [50]. Briefly, samples were automatically introduced into the sample chamber and the following script was used for EV measurements: PRIME, DELAY 5, CAPTURE 30, REPEAT 9. Samples were recorded using a camera level of 12 (camera shutter speed 15ms; camera gain 350) and NTA post-acquisition settings were optimised and kept constant between samples. Each video recording was analysed to give EV size and concentration measurements.

### Immunoblotting

Immunoblotting was performed as described previously [51]. For cell lysates, protein concentration was determined using a BCA protein assay kit (Pierce) and equal amounts of protein (10μg) were loaded. For EV preparations, equal volumes of the resuspended EV pellets were loaded. Primary antibodies were purchased from Abcam (β-actin (ab6276), HMGB1 (ab18256), syntenin (ab144267), Cited-2 (ab108345), total JNK (ab179461), phospho-JNK (ab179461) and Cell Signaling Technology (GRP78 (3183), CHOP (2985), cleaved PARP (9541), hsp70 (4872), histone H3 (4499), alix (2171), p-eIF2α (9721).

### Protein arrays

Protein arrays were performed using a Proteome Profiler Human Cell Stress Array Kit (R&D Systems). Briefly, BeWo cell lysates were incubated with the arrays at 4°C overnight and proteins were detected according to the manufacturer's instructions. Dots were detected using X-ray film and densitometric analysis was carried out using Image J software.

### Optiprep^™^ density gradient centrifugation

Separation of EVs according to their buoyant densities was carried out using the method of Tauro et al [23]. Briefly, EV pellets were isolated from the culture medium from three T75 flasks of confluent BeWo cells as described above. Pellets were pooled and resuspended in PBS (500μl) and overlaid onto 5-40% discontinuous iodixanol gradients (prepared from Optiprep™ 60% (w/v) aqueous iodixanol, Axis-Shield PoC). Ultracentrifugation was carried out at 150,000x*g* (maximum) for 18 h at 4°C. Fractions (12 × 1ml) were collected manually and NTA was carried out to determine the EV concentration of each fraction. To determine the density of each fraction the refractive index was measured using a hand-held refractometer (Mettler Toledo) and converted to density using a the following equation:

*ρ* = 3.4713*η* − 3.6393

where *ρ* = density and *η* = refractive index.

### Lactate dehydrogenase activity assay

Cell death was assessed by measurement of lactate dehydrogenase (LDH) release into the culture medium. LDH activity was determined using an LDH Cytotoxicity Colorimetric Assay Kit II (BioVision) according to the manufacturer's protocol. Briefly, culture medium was aspirated and spun at 3000 x g for 2 min at room temperature and 10 μl aliquots of the supernatant were transferred into a 96-well plate. LDH Reaction Mix (100 μl) was added and after incubation for 30 min absorbance at 450 nm was measured using a FLUOstar Optima plate reader (BMG Labtech). Results were expressed as the percentage of total cellular LDH (measured by lysis of cells for 30 min prior to collection of culture supernatants) released into the culture medium.

### Statistics

The data presented represent mean ± SEM of at least three separate experiments. Differences between treatment groups were analysed by one-way ANOVA or two-tailed Student's t-test, as indicated, and a *P*-value of < 0.05 was considered to be statistically significant.
